# Improving laboratory criteria to reduce unnecessary peripheral blood smear reviews by pathologists

**DOI:** 10.1093/labmed/lmag061

**Published:** 2026-09-17

**Authors:** Lincoln A Brown, Elizabeth Simonds, Claudio A Mosse

**Affiliations:** Department of Pathology, Microbiology, and Immunology, Vanderbilt University Medical Center, Nashville, TN, United States; Vanderbilt University School of Medicine, Nashville, TN, United States; Department of Medicine, Vanderbilt University Medical Center, Nashville, TN, United States; Department of Pathology, Microbiology, and Immunology, Vanderbilt University Medical Center, Nashville, TN, United States; Vanderbilt University School of Medicine, Nashville, TN, United States; Department of Pathology, Microbiology, and Immunology, Vanderbilt University Medical Center, Nashville, TN, United States

**Keywords:** complete blood cell count (CBC), peripheral blood smear, smear review, quality improvement, review criteria, flagging criteria

## Abstract

**Introduction:**

Pathologists frequently perform reviews of peripheral blood smears with limited clinical impact. There is considerable heterogeneity, however, in the laboratory criteria different institutions use to trigger these pathologist blood smear reviews (PBSRs).

**Methods:**

We retrospectively reviewed 1782 laboratory-initiated PBSRs performed at our institution to understand the indications for PBSR and their outcomes. Based on our findings, we revised our laboratory’s PBSR criteria as part of a quality improvement initiative to reduce the number of unnecessary reviews. All PBSRs were tracked for several months before and after the intervention to assess the impact of our intervention.

**Results:**

At baseline, 89% of PBSRs yielded no additional information, and only 6% yielded new information that appeared to be clinically impactful. The PBSR criteria based on abnormal platelet counts or leukopenia contributed to a disproportionate number of noncontributory reviews. Removing these conditions from our criteria led to an increase in the proportion of PBSRs yielding novel information from 7% to 16% and reduced the total number of reviews performed monthly by 17%.

**Discussion:**

Removal of low-yield conditions from our laboratory’s PBSR criteria led to a reduction in the number of unnecessary reviews performed. Our work demonstrates a practical quality improvement strategy to improve PBSR utility without compromising patient care.

## Introduction

**Figure 1:**
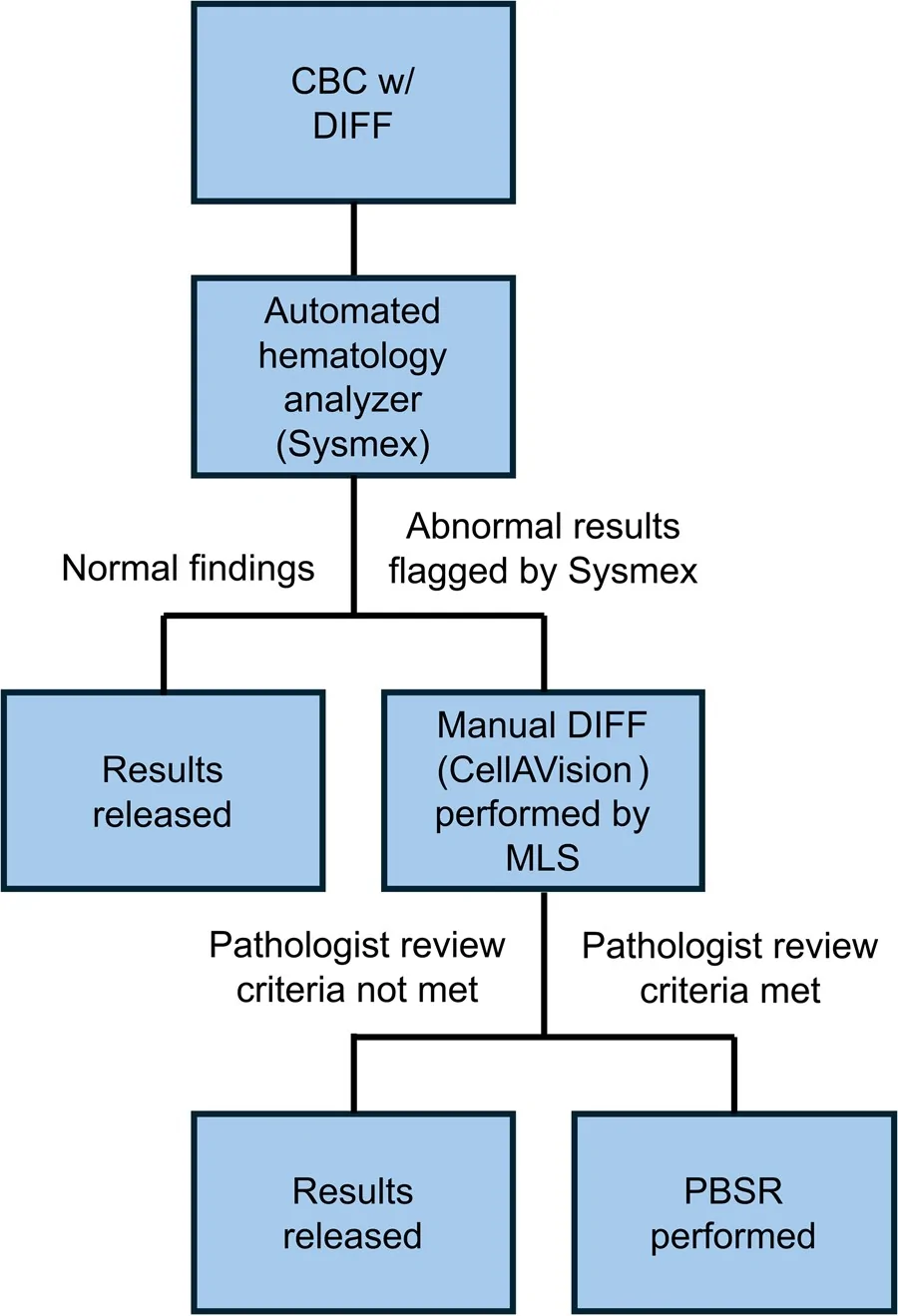
Laboratory workflow for complete blood cell count (CBC)/differential leukocyte count processing.

The complete blood cell count (CBC) and differential leukocyte count are among the most commonly performed tests in clinical laboratories. Modern automated hematology analyzers are capable of analyzing large numbers of samples and accurately and efficiently assessing cell counts and morphologic abnormalities, often with accuracy and precision comparable to manual screening.^1‐4^ Although the prevalence of these analyzers has reduced the need for human review, manual determinations of blood smear morphology and differential leukocyte counts by a trained morphologist remain important for evaluating abnormal results.^5^ A manual differential leukocyte count and preliminary morphologic examination are often performed by a trained technologist or medical laboratory scientist (MLS). In select cases, pathologists will perform a more thorough pathologist blood smear review (PBSR), integrating CBC and other laboratory values, morphologic findings, and clinical history. The PBSR serves to ensure accuracy of findings, inform diagnoses, and guide cost-effective workups; it can be ordered by clinicians or initiated based on laboratory-specific criteria. Our laboratory’s workflow (Figure 1) is similar to the workflow reported at peer institutions.^5‐7^

Laboratory criteria for PBSR can vary substantially between laboratories.^5^^,^^6^^,^^8‐11^ More than 20 years ago, the International Consensus Group for Hematology Review (ICGHR) published recommendations for follow-up of abnormal automated hematology analyzer CBC/differential leukocyte count results.^12^ More recently, an international Peripheral Smear Review Working Group (PSRWG) published updated recommendations and a practical framework for PBSR implementation.^13^ However, these guidelines largely focus on follow-up of abnormal automated analyzer results at the MLS level, with little guidance specifically on escalation for pathologist review. Pathologist blood smear reviews require substantial time and expertise, yet in many cases they do not provide added clinical value and, moreover, are not seen by physicians,^9^^,^^14^^,^^15^ highlighting the need to develop criteria to capture cases that require pathologist expertise while minimizing waste. Unsurprisingly, many pathologists have expressed a desire for improved or standardized review criteria.^7^

In this study, we undertook a quality improvement initiative to assess and improve the utility of laboratory-initiated PBSRs. We examined historical PBSR data at our institution and determined the frequencies and outcomes of reviews for different indications. Based on our findings, we revised our laboratory’s criteria for PBSR, which led to a reduction in the number of unnecessary reviews performed. Our study presents a strategy that institutions can use to optimize PBSR criteria and improve efficiency without compromising patient care.

## Methods

### Study design

At our institution, abnormal slide review forms are initiated by an MLS when a criterion is met for PBSR (Table 1). The MLS personnel record their interpretation and the indication for PBSR on the form, then send the form and the slide to a pathologist. In cases where a sample does not meet predefined criteria for PBSR but the MLS believes it should be reviewed by a pathologist, the MLS can mark “Other” on the abnormal slide review form and write in a reason for PBSR. The pathologist then reviews the slides, patient history, and reported CBC/differential leukocyte count and indicates on the form that they either agree with the MLS’s interpretation or disagree and provide a new or modified interpretation. We retrospectively reviewed all abnormal slide review forms from December 2023 to June 2025 (*n* = 1782 [approximately 3.9% of all flag-triggered MLS manual differentials, approximately 0.18% of all CBC/differential leukocyte counts). No distinction was made between inpatient and outpatient samples because both are subject to the same laboratory workflow. For each case, we recorded the indication for and the outcome of review. Our primary measures were total number of reviews and rate of change in interpretation of reviews for each PBSR indication. Interpretation change was defined as a revision or addition of new information to the MLS’s interpretation by the reviewing pathologist.

**Table 1:** PBSR criteria (pre-intervention).

**WBC abnormalities**
WBC count >30 000/μL (new patients)
WBC count <2000/μL (new patients)
Blasts >1%
Promyelocytes >20%^a^ or Auer rods
Atypical lymphocytes >10%
Large granular lymphocytes >10%
Cleaved lymphocytes
Plasma cells/plasmacytoid lymphocytes >5%
Large abnormal atypical cells
Abnormal inclusions
Hyposegmented or hypersegmented nuclei >5%
**Red blood cell abnormalities**
Schistocytes and decreased platelet count
Microspherocytes ≥2+
Abnormal inclusions (eg, malaria)
**Platelet abnormalities**
Platelet count <50 000/μL (new patients)
Platelet count >750 000/μL (new patients)
**Other**

Abbreviations: PBSR, pathologist blood smear review; WBC, white blood cell.

a The >20% promyelocyte threshold is a historical institutional criterion that does not reflect current diagnostic standards for acute promyelocytic leukemia.

As a secondary measure, we assessed whether interpretation changes were likely to be clinically impactful. Potential clinical impact was assessed based on the nature of the interpretation change and the clinical context. For example, a PBSR that identified abnormal cell morphologies (eg, blasts, schistocytes) that might inform new diagnoses or prompt further evaluation were considered likely to be clinically impactful. In addition, if pathologists reclassified a clinically significant abnormality, such as blasts, to something else, such as reactive or normal lymphocytes, we considered this likely to be clinically impactful because the original interpretation could have altered clinical management. In contrast, reviews that reidentified known abnormalities or added information based primarily on clinical history rather than morphology were considered unlikely to have a clinical impact. Examples from our data illustrating this classification scheme are shown in Table S1. Assessment of likely clinical impact was intended as a secondary exploratory measure due to greater subjectivity, limited availability of clinical data in our analysis, and low numbers for many indications. All abnormal slide review forms were initially reviewed and classified by 1 individual. Cases of unclear clinical impact were reviewed by a senior hematopathologist, who determined the final classification.

### Intervention

In June 2025, a new abnormal slide review form was introduced with revised PBSR criteria. To evaluate the effects of this intervention, we tracked all PBSRs before and after the intervention from November 2024 through September 2025, recording the monthly numbers of reviews performed and rates of interpretation change. Because this study was undertaken as a quality improvement effort, we did not have a control group. Instead, we used run charts and statistical process control (SPC) charts to analyze our intervention; SPC charts are commonly used analytical methods in quality improvement for evaluating changes in a process over time.^16‐18^

### Data analysis

Data were collected using Excel (Microsoft). Charts were prepared using Excel or Prism 11.0 software (GraphPad Software). All statistical analyses were performed as described in the text and figure legends using Prism or Excel.

## Results

The results of 1782 PBSRs performed between December 2023 and June 2025 are shown in Table 2. A PBSR was most commonly initiated for white blood cell (WBC) abnormalities (1146 [64%]), followed by platelet abnormalities (310 [17%]), “Other” (263 [15%]), and red blood cell abnormalities (63 [4%]). The presence of blasts (443 [25%]), atypical lymphocytes (332 [19%]), and low platelet counts (195 [11%]) were the most cited specific indications for PBSR. The most common reasons provided for PBSR in the “Other” category were to rule out malaria (part of a standard protocol for high-risk patients at our institution; 38%); MLS uncertainty regarding classification of potentially abnormal cells (33%); or presence of an abnormal cell population not included in PBSR criteria, such as prolymphocytes or hairy cells (19%) (Table S2).

**Table 2:** PBSR indications and outcomes.

Indication	Reviews, No. (%)^a^	No change, No. (%)^b^	Interpretation change, No. (%)^b^	Interpretation change with likely clinical impact, No. (%)^b^
**WBC abnormalities** ^c^	1146 (64)	1039 (91)	107 (9)	50 (4)
WBC count >30 000/μL (new patients)	131 (7)	129 (98)	2 (2)	1 (1)
WBC count <2000/μL (new patients)	64 (4)	64 (100)	0 (0)	0 (0)
Blasts >1%	443 (25)	394 (89)	49 (11)	30 (7)
Promyelocytes >20% or Auer rods	2 (0)	2 (100)	0 (0)	0 (0)
Atypical lymphocytes >10%	332 (19)	316 (95)	16 (5)	5 (2)
Large granular lymphocytes >10%	12 (1)	11 (92)	1 (8)	0 (0)
Cleaved lymphocytes	9 (1)	5 (56)	4 (44)	1 (11)
Plasma cells/plasmacytoid lymphocytes >5%	22 (1)	20 (91)	2 (9)	0 (0)
Large abnormal atypical cells	59 (3)	31 (53)	28 (47)	12 (20)
Abnormal inclusions	16 (1)	12 (75)	4 (25)	1 (6)
Hyposegmented or Hypersegmented nuclei >5%	56 (3)	55 (98)	1 (2)	0 (0)
**Red blood cell abnormalities** ^c^	63 (4)	59 (94)	4 (6)	4 (6)
Schistocytes and low platelet count	47 (3)	44 (94)	3 (6)	3 (6)
Microspherocytes ≥2+	12 (1)	11 (92)	1 (8)	1 (8)
Abnormal inclusion (eg, malaria)	4 (0)	4 (100)	0 (0)	0 (0)
**Platelet abnormalities** ^c^	310 (17)	310 (100)	0 (0)	0 (0)
Platelet count <50 000/μL (new patients)	195 (11)	195 (100)	0 (0)	0 (0)
Platelet count >750 000/μL (new patients)	115 (6)	115 (100)	0 (0)	0 (0)
**Other**	263 (15)	179 (68)	84 (32)	51 (19)
**Total**	**1782**	**1587 (89)**	**195 (11)**	**105 (6)**

Abbreviations: PBSR, pathologist blood smear review; WBC, white blood cell.

a Percentage among total PBSRs performed.

b Percentage among PBSRs performed for a given indication.

c Outcomes differed significantly between WBC, red blood cell, and platelet abnormalities (*P* < .001, Fisher exact test).

Overall, pathologists changed the MLS’s interpretation or added new information in 11% of cases. Rates of interpretation change varied greatly by criterion. There were statistically significant differences in the outcomes of PBSRs performed for WBC, red blood cell, and platelet abnormalities (*P* < .001; Fisher exact test), largely due to reviews for platelet abnormalities failing to yield new information in any cases. Among specific PBSR indications, the greatest rates of interpretation change were seen for large abnormal atypical cells (47%), cleaved lymphocytes (44%), and “Other” (32%). In cases of interpretation changes, we assessed whether changes were likely to be clinically impactful. Although a slight majority of interpretation changes appeared to be clinically impactful, the result amounted to just 6% of all PBSRs. Some criteria were notably more likely to yield impactful information, even if the overall frequency of interpretation change was relatively low. Among 443 PBSRs for blasts, 49 (11%) yielded changes in the interpretation, which were likely clinically impactful in 30 (7%) cases. In contrast, among 16 (5%) of 332 PBSRs for atypical lymphocytes resulting in interpretation changes, only 5 (2% overall) yielded likely clinically impactful information (Table 2). Notably, PBSRs in the “Other” category had high rates of interpretation change and likely clinical impact, particularly when performed due to MLS uncertainty regarding classification of abnormal cells (77% interpretation change; 50% with likely clinical impact) (Table S2).

**Figure 2:**
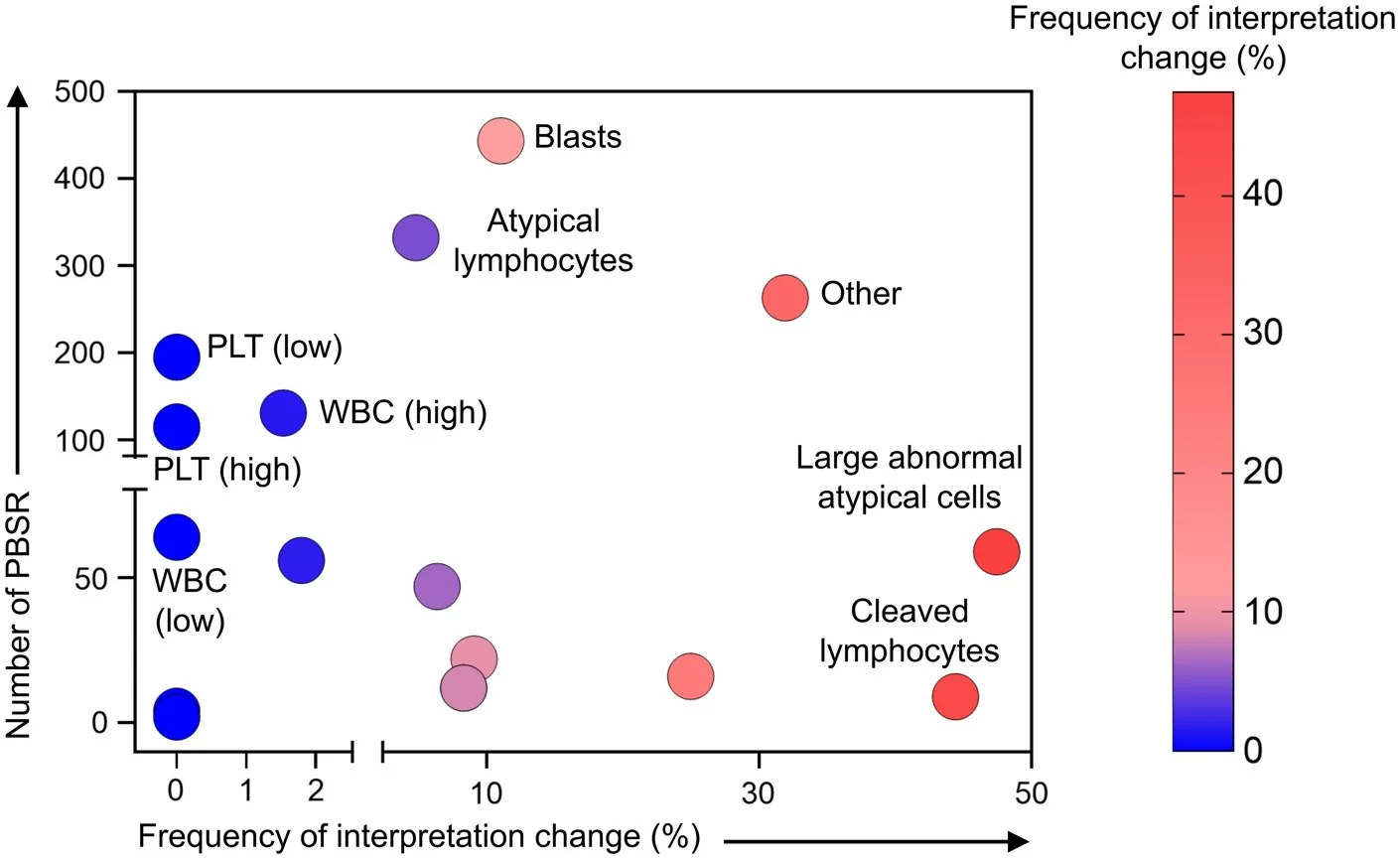
Total numbers and rates of interpretation change of reviews performed for each peripheral blood smear review criterion. Select criteria are labeled. PLT indicates platelet; WBC, white blood cell.

We sought to determine which criteria could be modified to improve the efficiency of our PBSR process. To evaluate the utility of individual PBSR indications, we considered the frequency with which a criterion was cited and the rate at which it yielded interpretation change or new information on pathologist review (Figure 2). Using this framework, we observed that criteria based solely on quantitative abnormalities generally had a lower yield than criteria based on qualitative or morphologic abnormalities. In particular, thrombocytosis, thrombocytopenia, and leukopenia contributed to a considerable number of PBSRs overall (combined 374 [21%]) but resulted in no instances of interpretation change or new information, suggesting that these criteria may be dispensable.

**Figure 3:**
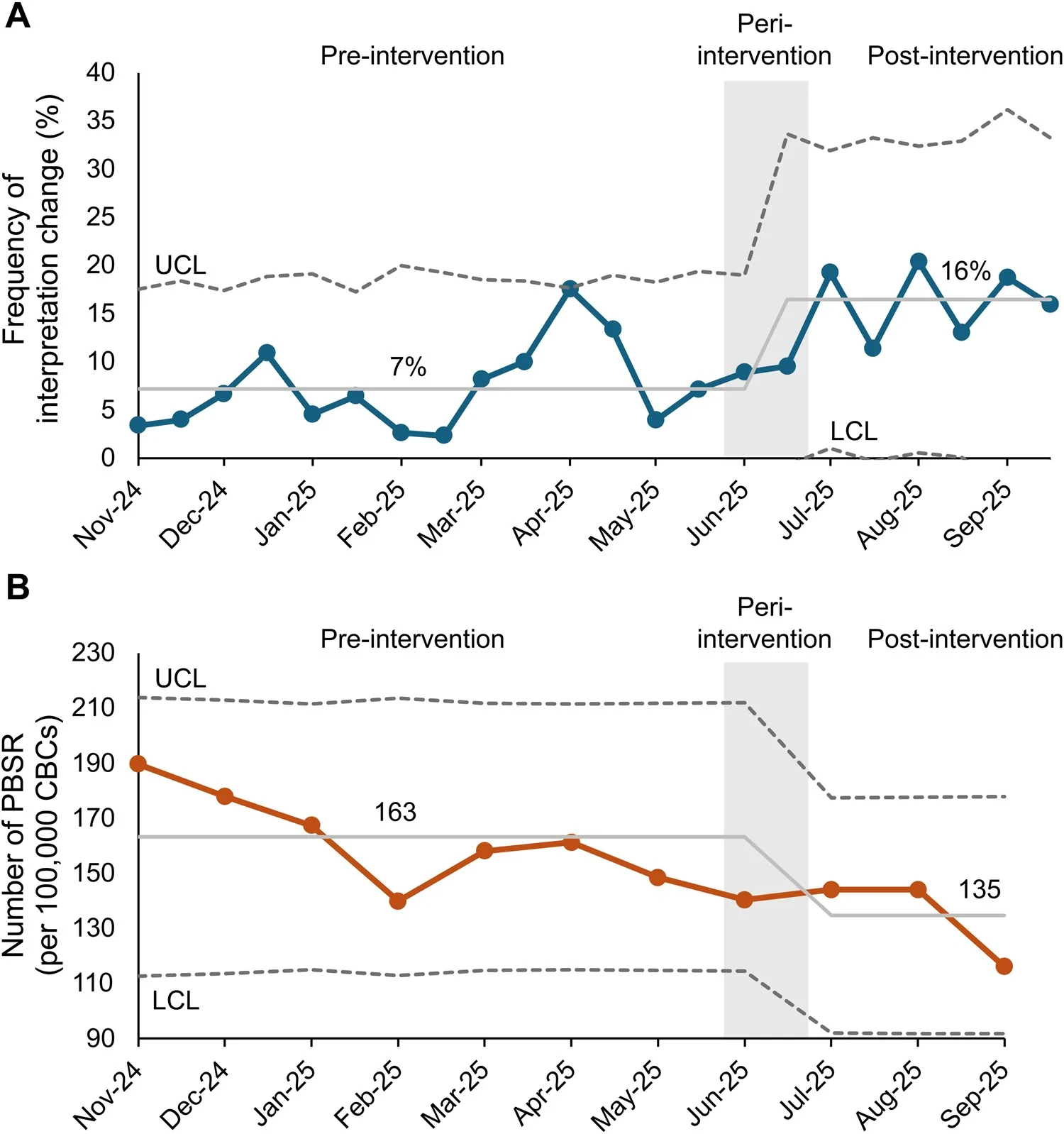
Statistical process control charts showing (**A**) rates of interpretation change and (**B**) numbers of pathologist blood smear reviews (PBSRs) over time. Center lines (gray lines) display mean values during preintervention or postintervention periods. Upper control limits (UCLs) and lower control limits (LCLs) (dashed lines) represent ±3 SDs from the centerline.

Based on these findings, we removed low platelet count, high platelet count, and low WBC count conditions from our laboratory’s PBSR criteria in June 2025 (Table 3). We tracked the rates of interpretation change and incidences of PBSRs monthly from November 2024 to September 2025, shown as SPC charts in Figure 3. After our intervention, the frequency of PBSRs that resulted in interpretation changes or new information increased from 7% to 16%. This change represented a nonrandom shift based on the frequency and degree of values above the center line after our intervention.^17^ In addition, the monthly number of PBSRs performed (per 100 000 CBCs) decreased by 17%. These results suggest that our intervention led to a reduction in the number of unnecessary PBSRs performed during the study period.

**Table 3:** PBSR criteria (post-intervention).

**WBC abnormalities**
WBC >30 000/μL (new patients)
Blasts >1%
Promyelocytes >20% or Auer rods
Atypical lymphocytes >10%
Large granular lymphocytes >10%
Cleaved lymphocytes
Plasma cells/plasmacytoid lymphocytes >5%
Large abnormal atypical cells
Abnormal inclusions
Hyposegmented or hypersegmented nuclei >5%
**Red blood cell abnormalities**
Schistocytes and decreased platelet count
Microspherocytes ≥2+
Abnormal inclusions (eg, malaria)
**Other**

Abbreviations: PBSR, pathologist blood smear review; WBC, white blood cell.

## Discussion

Modern laboratory workflows and instrumentation have reduced the need for humans in the processing of peripheral blood samples. The original ICGHR consensus recommendations and recent PSRWG recommendations provide guidance on manual follow-up of automated analyzer results at the MLS level.^13^ However, there is a lack of guidelines or evidence specifically addressing pathologist escalation, and there is substantial heterogeneity in PBSR criteria between laboratories.^5^^,^^6^^,^^12^^,^^15^ At our institution, we found that many pathologist reviews did not add novel information to the CBC/differential leukocyte count results, and an even lower number yielded clinically useful information, consistent with prior studies showing that PBSRs frequently add little clinical value yet require substantial time and expertise.^7^^,^^9^^,^^14^ Still, PBSRs can be an essential tool for accurate and efficient diagnosis and treatment, as evidenced by several cases in our data in which PBSRs contributed novel and seemingly clinically impactful information, such as the identification or reclassification of abnormal cell morphologies.

These findings highlight a need to optimize laboratory workflows to adequately capture cases in which pathologist expertise is clinically indicated while minimizing unnecessary reviews. Previous studies have suggested strategies to reduce the number of unnecessary clinician-ordered PBSRs, including modifying the electronic ordering procedure and implementing selective ordering criteria.^6^^,^^19^^,^^20^ However, few studies have explicitly evaluated laboratory-initiated protocols for PBSR.

To this end, we evaluated the laboratory-initiated PBSR criteria at our institution. We observed differences in the rates at which different criteria trigger PBSRs and contribute new information. Notably, we found that criteria based on quantitative WBC or platelet count cutoffs, in the absence of morphologic abnormalities, triggered a substantial number of PBSRs but rarely added new or clinically useful information. A similar finding was observed in a 3-institution study from Beckman et al,^21^ with reviews for platelet abnormalities or leukopenia showing low clinical utility, although they focused solely on clinician-ordered PBSRs. Moreover, although the recent PSRWG recommendations are targeted to smear reviews at the MLS level, they suggest considering tightening or eliminating several of the original ICGHR-recommended quantitative WBC or platelet count cutoffs; this recommendation agrees with our finding that these quantitative criteria have low yield at the pathologist level.^13^ Based on published review criteria from other institutions, the inclusion of quantitative cutoffs for these measures in PBSR criteria appears to vary between institutions.^5^^,^^6^^,^^11^

We aimed to improve our laboratory’s workflow by removing criteria that led to many noncontributory PBSRs. We removed criteria for high platelet count, low platelet count, and low WBC count, all of which failed to contribute any PBSRs of value in our analysis. After implementation, we noted an immediate reduction in the number of unnecessary PBSRs performed, as evidenced by an increase in the rate of reviews yielding novel information and a reduction in the overall number of PBSRs over the following months. As a rough estimate of operational impact, approximately 1 hour per day is spent on PBSRs at our institution, translating to roughly $50 per day saved through a 17% reduction in review time (including both pathologist and technologist time), although this time is variable by pathologist practice and laboratory PBSR criteria. Our results demonstrated that optimizing PBSR criteria can be an effective strategy for reducing waste.

Additional criteria may also be considered for modification or removal to improve PBSR value. High WBC count and atypical lymphocytes, for example, were among the most common criteria triggering PBSR, yet PBSR contributed new information in only about 2% and 5% of cases, respectively. Although we found that pathologist reviews of these cases often simply confirmed the presence of the abnormality in a patient with a consistent clinical history, they rarely identified or raised concern for more clinically significant abnormalities, such as blasts or abnormal (neoplastic) lymphocytes. In these cases, the benefits of identifying a small number of impactful cases must be weighed against the burden of reviewing many uninformative smears. For our initial intervention, we made the decision to keep these criteria unchanged due to the risk of missing those few cases with clinically significant findings.

The risk of false negatives, or samples with missed clinically significant findings that fail to reach the level of pathologist review because they did not meet PBSR criteria, is important to consider when designing or modifying PBSR criteria. To minimize false negatives, PBSR criteria should capture cases in which MLSs might miss or incorrectly classify a clinically significant finding. A limitation of our study is that we were unable to measure false-negative rates because we analyzed only cases reviewed by pathologists. Reassuringly, our preintervention data showed that the 3 removed criteria failed to trigger any PBSR that contributed new or clinically significant information over the previous 18 months, suggesting that their removal was unlikely to lead to an increase in false negatives or negatively affect patient care.

Although prior studies have examined false-negative rates for automated analyzer cutoffs,^22^^,^^23^ there are few data on false negatives at the pathologist level. Although we were unable to measure false negatives in our study, our data suggest that certain PBSR criteria are more useful for mitigating false negatives. For example, we observed that PBSRs for many abnormal WBC morphologies, such as blasts and neoplastic lymphocytes, led to higher rates of interpretation change, suggesting that modifying these criteria carries a greater risk of introducing false negatives. Our PBSR criteria also include a flexible “Other” category, which allows MLSs to escalate a smear to pathologist review if they are unsure of a finding. A PBSR requested because of MLS uncertainty frequently led to new and likely clinically impactful information, suggesting that inclusion of a discretionary escalation pathway is valuable and can help mitigate false negatives. Substantial changes to PBSR criteria, particularly those that occasionally aid in the identification of clinically significant findings, should be validated to ensure that false-negative rates remain low. The recent PSRWG recommendations, although focused on smear review at the MLS level, provide a useful framework for designing validation studies that can also be applied to PBSR criteria.^13^ Future quality improvement cycles will examine and validate additional changes to PBSR criteria that could improve value.

There were other limitations to our study, as well. Our intervention was undertaken as a quality improvement effort, and causality cannot be definitively established given the absence of a comparator or control group. Nonetheless, we applied validated statistical methods commonly used in quality improvement work; these methods demonstrated nonrandom shifts in our data that strongly suggest an effect from our intervention. We believe that our data and framework can help inform future efforts in our and other laboratories to improve PBSR efficiency.

In addition, our study focused only on PBSR protocols at our institution, which may differ from other settings with respect to laboratory protocols, populations served, experience and training of medical staff, and volume of work. In particular, differences in MLS availability and level of training likely affect PBSR yield and should be considered when designing effective PBSR criteria.^7^^,^^13^ Our findings are most directly generalizable to peer academic institutions, which feature similar workflows and supporting staff.^6^^,^^7^ However, smaller practices with less MLS support are more likely to deal with a greater burden of pathologist reviews, making it valuable in these settings, as well, to identify strategies to reduce waste. We recommend that other institutions consider our findings and tailor PBSR criteria to their individual needs to best support pathologists and clinicians. In particular, we found that PBSR criteria based on quantitative platelet and WBC abnormalities contribute to a disproportionate number of noncontributory reviews, and removal of these criteria may help reduce the number of unnecessary PBSRs performed. Future work will evaluate additional low-impact criteria as well as examine other areas that may contribute to inefficient CBC processing, such as indications for MLS manual differential and review based on automated hematology analyzer results. Additional studies at multiple sites may help define standardized criteria or recommendations for PBSR.

## Supplementary Material

lmag061_Supplementary_Data

## Data Availability

All data generated and supporting the findings of this study are available in the paper or are available from the corresponding author on request.
