# Supplementary material for: Improving laboratory criteria to reduce unnecessary peripheral blood smear reviews by pathologists
Source: Lab Med. 2026 Sep 17;57(5):lmag061. doi: 10.1093/labmed/lmag061 (PMC13585456; doi:10.1093/labmed/lmag061)
Supplement: Supplementary file 1 [file lmag061_supplementary_data.zip › labmed-2026-05-u-sci-0193-File009.docx]

**Supplementary Table S2** Indications and outcomes of PBSR in “Other” category

| Indication | Number of reviews (%)^a^ | No change (%)^b^ | Interpretation change (%)^b^ | Interpretation change, likely clinical impact (%)^b^ |
| --- | --- | --- | --- | --- |
| Malaria rule-out | 100 (38) | 98 (98) | 2 (2) | 2 (2) |
| MLS uncertain of classification / requesting clarification | 87 (33) | 20 (23) | 67 (77) | 43 (50) |
| Other abnormal cells not included in PBSR criteria (e.g. prolymphocytes, hairy cells) | 51 (19) | 39 (76) | 12 (24) | 4 (8) |
| Other / no reason given | 25 (10) | 22 (88) | 3 (12) | 2 (8) |
| **Total** | **263** | **179 (68)** | **84 (32)** | **51 (19)** |

^a^Percent among total “Other” PBSR performed

^b^Percent among PBSR performed for given indication

Abbreviations: MLS, medical laboratory scientist; PLT, platelets; WBC, white blood cells
