# Supplementary material for: Improving laboratory criteria to reduce unnecessary peripheral blood smear reviews by pathologists
Source: Lab Med. 2026 Sep 17;57(5):lmag061. doi: 10.1093/labmed/lmag061 (PMC13585456; doi:10.1093/labmed/lmag061)
Supplement: Supplementary file 1 [file lmag061_supplementary_data.zip › labmed-2026-05-u-sci-0193-File008.docx]

**Supplementary Table S1** Select examples of PBSR outcomes

**No change in interpretation**

- Technologist called “3.4% blasts” and sent slide for PBSR. On review, pathologist noted “agree” and did not alter interpretation

**Change interpretation**

**Unlikely clinical impact**

- Technologist cited “atypical lymphocytes”. On review, pathologist reclassified cells as abnormal lymphocytes in patient with known T-cell prolymphocytic leukemia.
- Technologist cited “large abnormal atypical cells”. On review, pathologist classified cells as reactive lymphocytes in child with sepsis.
- Technologist cited “cleaved lymphocytes”. On review, pathologist classified cells as abnormal lymphocytes in patient with mantle cell lymphoma.

**Likely clinical impact**

- Technologist cited “atypical lymphocytes”. On review, pathologist reclassified as “15% blasts” in patient with no history of blasts.
- Technologist cited “large abnormal atypical cells”. On review, pathologist classified cells as blasts, ordered flow cytometry to correlate, and alerted physician.
- Technologist cited “blasts”. On review, pathologist stated “no definitive blasts—reactive lymphs + a few hypogranular neutrophils”.
- Technologist cited “schistocytes and decreased platelet count”. On review, pathologist disagreed with schistocyte classification and removed schistocytes from interpretation.
